# Antidiabetic Effect of Collagen Peptides from *Harpadon nehereus* Bones in Streptozotocin-Induced Diabetes Mice by Regulating Oxidative Stress and Glucose Metabolism

**DOI:** 10.3390/md21100518

**Published:** 2023-09-29

**Authors:** Qianxia Lin, Yueping Guo, Jie Li, Shuqi He, Yan Chen, Huoxi Jin

**Affiliations:** 1 Zhejiang Provincial Engineering Technology Research Center of Marine Biomedical Products, School of Food and Pharmacy, Zhejiang Ocean University, Zhoushan 316022, China; linqianxia@zjou.edu.cn (Q.L.);; 2 Jinhua Food and Drug Inspection and Testing Institute, Jinhua 321015, China

**Keywords:** diabetes, collagen peptides, Nrf2, glucose metabolism, oxidative stress

## Abstract

Oxidative stress and abnormal glucose metabolism are the important physiological mechanisms in the occurrence and development of diabetes. Antioxidant peptides have been reported to attenuate diabetes complications by regulating levels of oxidative stress, but few studies have focused on peptides from marine bone collagen. In this study, we prepared the peptides with a molecular weight of less than 1 kD (HNCP) by enzymolysis and ultrafiltration derived from *Harpadon nehereus* bone collagen. Furthermore, the effects of HNCP on blood glucose, blood lipid, liver structure and function, oxidative stress, and glucose metabolism were studied using HE staining, kit detection, and Western blotting experiment in streptozocin-induced type 1 diabetes mice. After the 240 mg/kg HNCP treatment, the levels of blood glucose, triglyceride (TG), and low-density lipoprotein cholesterol (LDL-C) in streptozotocin-induced diabetes mice decreased by 32.8%, 42.2%, and 43.2%, respectively, while the levels of serum insulin and hepatic glycogen increased by 142.0% and 96.4%, respectively. The antioxidant enzymes levels and liver function in the diabetic mice were markedly improved after HNCP intervention. In addition, the levels of nuclear factor E2-related factor 2 (Nrf2), glucokinase (GK), and phosphorylation of glycogen synthase kinase-3 (p-GSK3β) in the liver were markedly up-regulated after HNCP treatment, but the glucose-6-phosphatase (G6Pase) and phosphoenolpyruvate carboxykinase1 (PEPCK1) were down-regulated. In conclusion, HNCP could attenuate oxidative stress, reduce blood glucose, and improve glycolipid metabolism in streptozocin-induced type 1 diabetes mice.

## 1. Introduction

Diabetes mellitus is a syndrome characterized by the disorder of sugar and lipid metabolism. Diabetes is known as a “silent killer” due to a large number of chronic complications [1,2]. Low levels of in insulin in the body and a decline in glucose metabolism will lead to an increase in blood glucose [3]. At present, some enzymes related to glucose metabolisms, such as glucokinase (GK) [4], phosphoenolpyruvate carboxykinase1 (PEPCK1) [5], and glucose-6-phosphatase (G6Pase) [6], have been confirmed to be associated with diabetes. Under long-term high hyperglycemia, a large number of reactive oxygen species (ROS) are accumulated in the body, resulting in cell damage and tissue dysfunction. The liver is damaged due to the long-term accumulation of hyperglycemia [7], which can cause metabolic abnormalities and dysfunction, and eventually lead to non-alcoholic fatty liver and other complications [8,9]. Studies have shown that diabetes and its complications are closely related to oxidative stress and glucose metabolism caused by high glucose, but the mechanism is still unclear [10,11].

Because oxidative stress is closely related to diabetes, one of the potential strategies for preventing and treating diabetes is to reduce oxidative stress levels [12]. Studies have reported that some bioactive peptides have dual antioxidation and hypoglycemic functions, such as peptides from yeast hydrolysates [13], milk protein-derived hydrolysates [14], and egg-yolk protein hydrolysates [15]. In the past decades, bioactive peptides from marine organisms have attracted extensive attention due to their various biological properties, including antioxidant [16,17,18], antihypertensive [19], antidiabetic [20], immunoregulation [21], and antifatigue [22] properties. The Bombay duck (Harpadon nehereus), an important edible fish in China, is widely distributed in the coastal areas of China. The meat of Harpadon nehereus is soft, tender and smooth, and rich in protein (up to 70% of the dry weight) [23]. The bones of the Harpadon nehereus are discarded because they are not edible, but they are rich in collagen and calcium, causing a waste of resources. Therefore, the preparation of collagen peptides with nutritional or medical value from *Harpadon nehereus* bones can greatly improve the economic value of *Harpadon nehereus*.

Nuclear factor E2-related factor 2 (Nrf2) plays a backbone role in cellular antioxidant defense. Nrf2 regulates the expression of antioxidant enzyme genes by binding to anti-response elements (ARE) [24]. In addition to Nrf2, the ARE binding site is also the target gene for NAD(P)H quinone oxidoreductase 1 (NQO1) and heme-oxygenase (HO-1) [25]. The activation of the Nrf2-ARE signal pathway has been shown to reduce the production of free radicals and oxidative stress, playing a protective role in the kidney [26,27]. However, it has not been elucidated yet whether collagen peptides from Harpadon nehereus bones have preventive and therapeutic effects on streptozocin-induced diabetes. Therefore, the purpose of this study was to evaluate the therapeutic effect and explore the potential mechanism of collagen peptides from Harpadon nehereus in streptozocin-induced diabetic mice, laying a theoretical foundation for the application of collagen peptides in diabetes prevention.

## 2. Results

### 2.1. Preparation of HNCP and Its Antioxidative Activity

**Figure 1:**
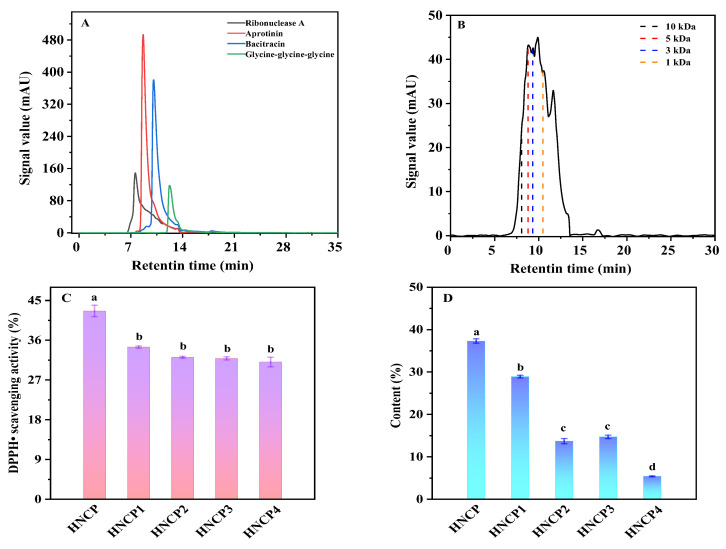
The HPLC diagram of standards (**A**) and hydrolysates of bone collagen from *Harpadon nehereus* (**B**); The DPPH• scavenging activity (**C**) and content (**D**) of each component in the hydrolysates. Values with different letters (a–d) indicate significant differences between groups (*p* < 0.05).

The hydrolysates of bone collagen from *Harpadon nehereus* were analyzed by high-performance liquid chromatography (HPLC) with chromatographic column TSK-GEL 2000SWXL. The ribonuclease A, aprotinin, bacitracin, and glycine-glycine-glycine were used as the standard substances. As shown in Figure 1A, all the standard substances were eluted within 20 min. According to the relationship between the logarithm of relative molecular mass (lg Mw) and retention time (t) of each standard, the standard curve was obtained: lg Mw = −0.4105t + 7.3036, R2 = 0.997. The molecular weight logarithm showed a good correlation with the retention time under the chromatographic conditions. Thus, the standard curve was used to evaluate the molecular weight distribution of peptides in the hydrolysates of the bone collagen from *Harpadon nehereus*. Figure 1B shows that the molecular weight (Mw) of the hydrolysates by protease was mostly distributed below 3 kDa. According to the range of the molecular weight, the peptides were divided into five components, which were respectively named HNCP (Mw < 1 kDa), HNCP 1 (1~3 kDa), HNCP 2 (3~5 kDa), HNCP 3 (5~10 kDa), and HNCP 4 (Mw > 10 kDa). The content and DPPH• scavenging rate of HNCP were 37.3% and 44.1%, respectively, both of which were the highest among all components (Figure 1C,D). The amino acid analysis (Table 1) showed that HNCP was rich in glycine (Gly, 336.2 residues), alanine (Ala, 117.3 residues), and proline (Pro, 116.0 residues) but low in hydrophobic amino acids such as phenylalanine (Phe, 12.3 residues), isoleucine (Ile, 12.3 residues), and tyrosine (Tyr, 4.2 residues). In addition, the contents of hydroxyproline (Hyp) and glutamic acid (Glu) were more than 70 residues/1000 residues.

### 2.2. Effects of HNCP on Glucose Metabolism in Diabetic Mice

**Figure 2:**
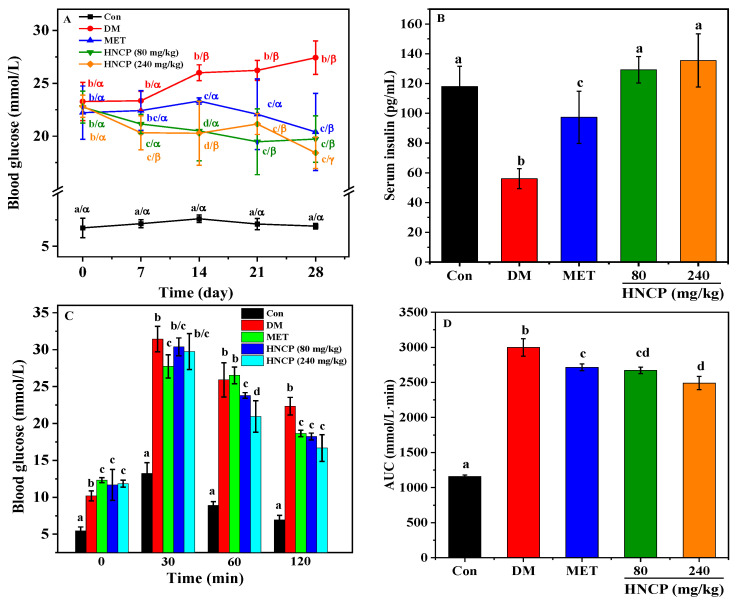

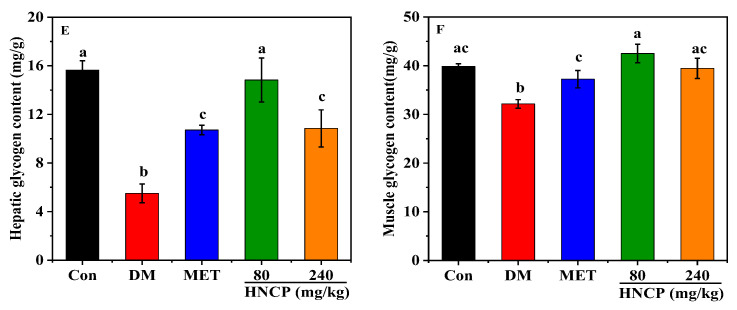
Effects of HNCP treatments on blood glucose (**A**), serum insulin (**B**), glucose tolerance (**C**,**D**), and glycogen levels (**E**,**F**) in STZ-induced type 1 diabetic mice. Values with different letters (a–d) indicate significant differences between groups at the same time (*p* < 0.05); Values with different letters (α–γ) indicate significant differences between different times in the same group (*p* < 0.05).

The intervention effect of HNCP on glucose metabolism in STZ-induced type 1 diabetic mice was investigated. The blood glucose of mice in the Con group (the normal mice group) remained at a normal level all the time and was markedly lower than that of diabetic mice. After intervention with HNCP as shown in Figure 2A, the blood glucose levels gradually decreased and were significantly lower than those in the DM group (STZ-induced model group). Compared with that in the Con group, the serum insulin content in the DM group was significantly decreased (*p* < 0.05). Remarkably, the serum insulin levels of diabetic mice after HNCP intervention increased significantly to the level of the Con group (Figure 2B), but there was no significant difference between the 80 mg/kg and 240 mg/kg HNCP groups.

The oral glucose tolerance test was used to measure glucose tolerance in the diabetic mice, which can evaluate the secretory function of pancreatic β cells and reflect the ability to regulate blood glucose. After glucose intragastric administration, blood glucose rose rapidly to the highest levels in all groups and then declined (Figure 2C). At 120 min, the blood glucose levels in the Con group returned to normal but remained at a high level in the diabetic mice. However, high levels of blood glucose in the diabetic mice were significantly decreased by the HNCP intervention. Compared with the Con group, AUC increased significantly in the DM group (Figure 2D). The AUC value of the HNCP group was significantly lower than that of the DM group (*p* < 0.05). However, there was no significant difference in the AUC values of different doses of HNCP.

Glucose is mainly stored in the body as glycogen, which maintains the stability of blood glucose by synthesis or decomposition. Glycogen contents in diabetic mice were analyzed, and the results are shown in Figure 2E,F. Lower levels of liver and muscle glycogen were observed in the DM group relative to that of the Con group (*p* < 0.05). It was observed that the glycogen levels of two organs were significantly increased (*p* < 0.05) after metformin (MET) and HNCP treatments. However, the high-dose HNCP (240 mg/kg) group did not show higher glycogen levels than the low-dose (80 mg/kg) HNCP group. These results indicate that HNCP can promote glycogen synthesis in diabetic mice and increase the ability of glucose uptake in the blood.

### 2.3. Effects of HNCP on Blood Lipids in Diabetic Mice

**Figure 3:**
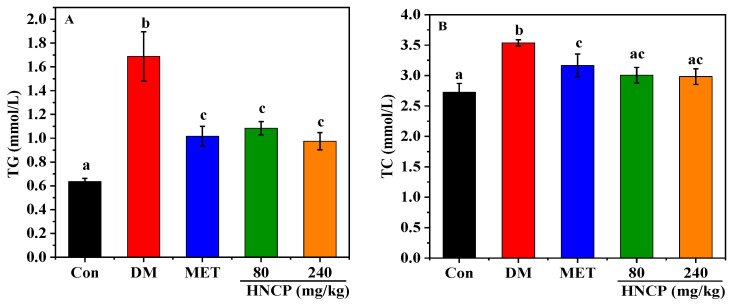

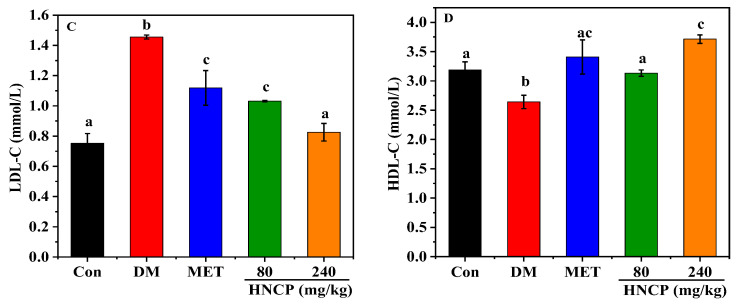
Effects of HNCP treatments on the contents of TG (**A**), TC (**B**), LDL-C (**C**), and HDL-C (**D**) in serum of STZ-induced type 1 diabetic mice. Values with different letters (a–c) represent significant differences between groups (*p* < 0.05).

The liver plays an important role in all stages of lipid metabolism, synthesis, and transport. Diabetes often leads to impaired liver function, which leads to dyslipidemia and accelerates liver damage. To investigate the improvement effects of HNCP on dyslipidemia, the contents of triglyceride (TG), total cholesterol (TC), high-density lipoprotein cholesterol (HDL-C), and low-density lipoprotein cholesterol (LDL-C) in serum were measured. As depicted in Figure 3A–D, serum TG, TC, and LDL-C contents in the DM group were significantly increased compared with the Con group (*p* < 0.05), while HDL-C content was significantly decreased (*p* < 0.05). HNCP intervention reversed the change trends of serum TG, TC, LDL-C, and HDL-C in the diabetic mice. In particular, the levels of TC, LDL-C, and HDL-C in the high-dose (240 mg/kg) HNCP group returned to the same levels as those in the Con group. There were significant differences in LDL-C and HDL-C levels between the two concentrations of HNCP groups, showing a concentration-dependent relationship, but no such relationship was observed for TC and TG levels. These results suggest that HNCP can effectively mitigate dyslipidemia in diabetic mice.

### 2.4. Improvement of HNCP on Liver and Pancreas Injury in Diabetes Mice

**Figure 4:**
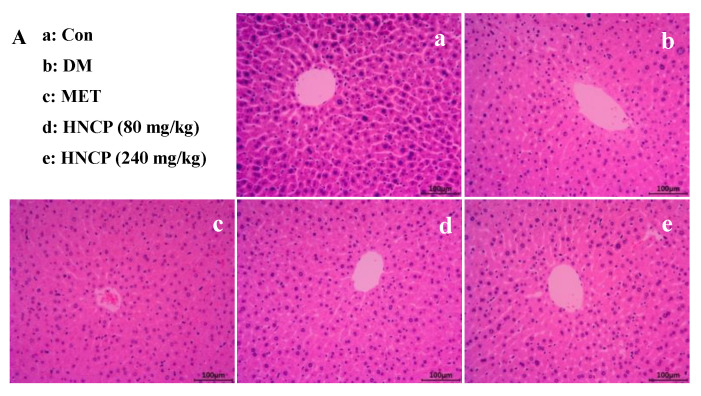

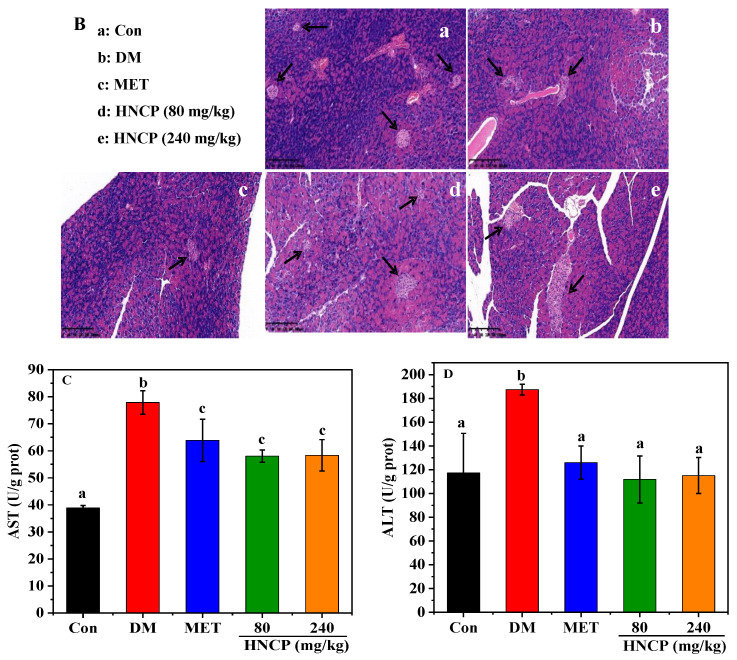
Effects of HNCP treatments on the liver structure (**A**, 200×), pancreas structure (**B**, 100×), and the levels of AST (**C**) and ALT (**D**) in STZ-induced type 1 diabetic mice. Different lowercase English letters (a–c) in (**C**,**D**) represent significant differences between groups (*p* < 0.05).

H&E staining in the liver and pancreas was performed to investigate the effects of HNCP treatment on the histological alterations of the liver and pancreas. As shown in Figure 4A, cells in the Con group were of a regular shape with clear boundaries, but more serious necrosis was observed in the DM group relative to that of the Con group. Meanwhile, hepatocytes were swollen and disordered, and some hepatocytes around the central vein were vacuolated in the DM group. However, it was observed that the adipose vacuoles and swelling of hepatocytes were significantly alleviated after MET and HNCP administration. Pancreatic islets (black arrow in Figure 4B) in the pancreas of mice in the Con group were normal in shape, clear in outline, and abundant in quantity. In the DM group, the volume and morphology of pancreatic islets were clearly changed. Blurred outlines, scattered structures, and a reduced number of pancreatic islets were observed. After the intervention of HNCP, the shape of pancreatic islets changed regularly, and the outline became clearer relative to that in the DM group.

Aspartate aminotransferase (AST) and alanine aminotransferase (ALT) are important indicators of liver function. To determine the improvement effects of HNCP treatment on liver function in the diabetic mice, the levels of plasma AST and ALT were measured (Figure 4C,D). AST and ALT levels in the DM group were observed to be higher than those of the Con group (*p* < 0.05). The high levels of ALT and AST were markedly decreased after MET and HNCP treatments, but there was no significant difference between the groups. It can be seen from the above results that HNCP can alleviate the liver injury of STZ-induced diabetic mice.

### 2.5. Effects of HNCP on Hepatic Oxidative Damage in Diabetic Mice

**Figure 5:**
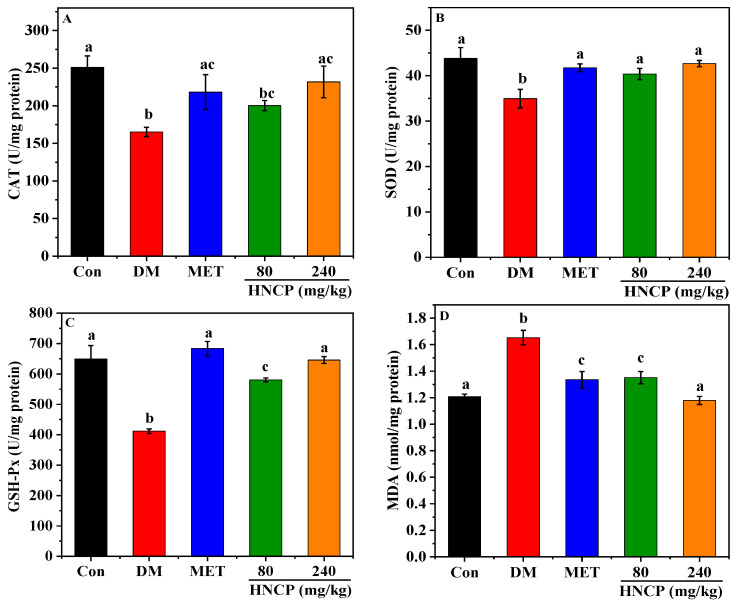
Effects of HNCP treatments on the levels of CAT (**A**), SOD (**B**), GSH-Px (**C**), and MDA (**D**) in the liver of STZ-induced type 1 diabetic mice. Values with different letters (a–c) represent significant differences between groups (*p* < 0.05).

According to the above experiments, HNCP has a free-radical scavenging ability in vitro. The effects of HNCP on the activities of catalase (CAT), superoxide dismutase (SOD), and glutathione peroxidase (GSH-Px), as well as the content of malondialdehyde (MDA), were determined to investigate whether HNCP can alleviate the oxidative stress level in diabetic mice. As shown in Figure 5A–D, the activities of CAT, SOD, and GSH-Px in the DM group were significantly lower than those in the Con group (*p* < 0.05), while the content of MDA was significantly higher (*p* < 0.05). Meanwhile, the activities of CAT, SOD, and GSH-Px were remarkedly increased in the HNCP groups in comparison with the DM group. The levels of CAT, SOD, GSH-Px, and MDA in the 240 mg/kg HNCP group were similar to those in the Con group. These results suggested that HNCP could effectively alleviate oxidative stress in the liver of STZ-induced type 1 diabetic mice.

### 2.6. Effects of HNCP on Nrf2 Signaling Pathway

**Figure 6:**
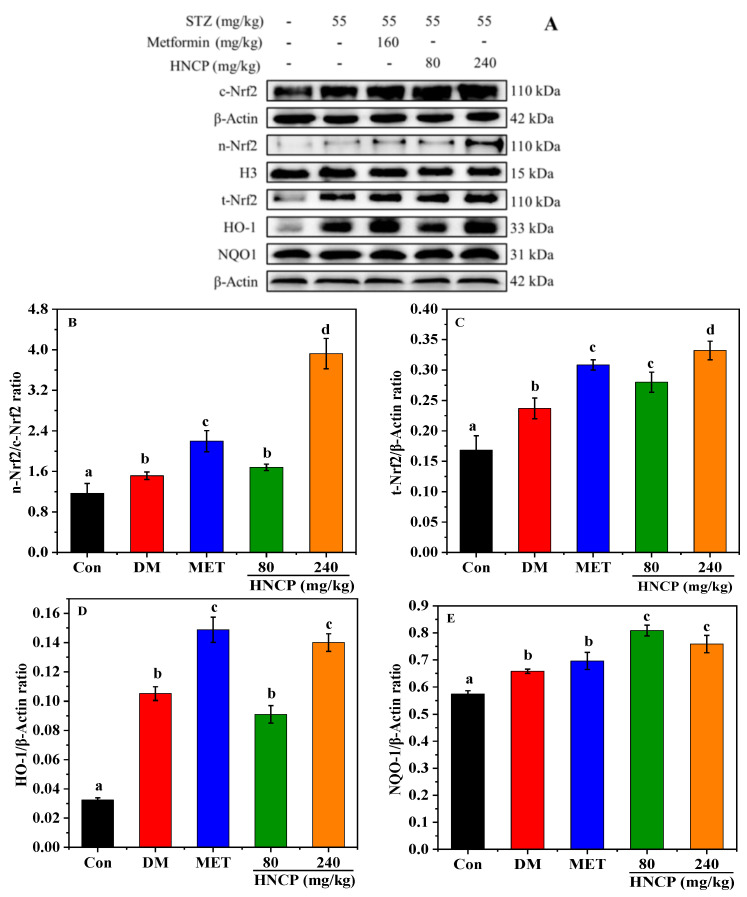
Effects of HNCP treatments on the expression of Nrf2 signaling pathway-related proteins in the liver of the STZ-induced diabetic mice (**A**). Analysis of protein expression levels of n-Nrf2/c-Nrf2 (**B**), t-Nrf2 (**C**), HO-1 (**D**), and NQO1 (**E**). Values with different letters (a–d) represent significant differences between groups (*p* < 0.05).

Nrf2 is an important endogenous transcription factor for cells to resist oxidative stress, which can regulate the expression levels of antioxidant genes such as heme oxygenase (HO-1) and NAD(P)H: quinone oxidoreductase 1 (NQO1). To elucidate the potential mechanism underlying HNCP-mediated alleviation of oxidative stress, the levels of Nrf2 and its related proteins (NQO1 and HO-1) expressions in the liver of STZ-induced diabetic mice were assessed by Western blot. As shown in Figure 6A–E, the levels of nuclear Nrf2 (n-Nrf2) in the DM group had no significant difference compared with the Con group, but the 240 mg/kg HNCP treatment significantly enhanced n-Nrf2 expression relative to that in the DM group (*p* < 0.05). Furthermore, the level of total Nrf2 (t-Nrf2) in the 240 mg/kg HNCP group was significantly higher than that in the DM group. The expression levels of downstream target proteins HO-1 and NQO1 in the 240 mg/kg HNCP group were significantly higher than those in the DM group, which was consistent with the n-Nrf2 level. These results indicated that HNCP might promote the transcription of Nrf2 into the nucleus to activate the Nrf2 signaling pathway, thereby increasing the expression of antioxidant enzymes such as HO-1 and NQO1.

### 2.7. Effects of HNCP on the Expression of Glucometabolic-Related Proteins

**Figure 7:**
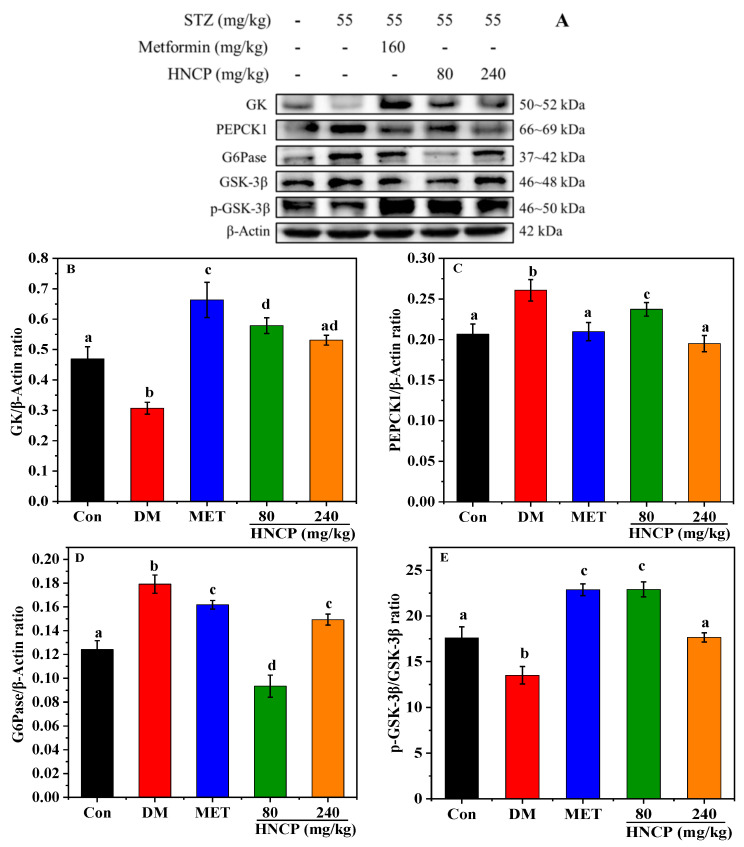
Effects of HNCP treatments on the expression of glucose metabolism-related proteins in STZ-induced type 1 diabetic mice (**A**). Analysis of GK (**B**), PEPCK1 (**C**), G6Pase (**D**), and p-GSK-3β (**E**) levels. Different letters (a–d) represent significant differences between groups (*p* < 0.05).

Glucokinase (GK), phosphoenolpyruvate carboxykinase1 (PEPCK1), and glucose-6-phosphatase (G6Pase) are key enzymes in glucose metabolism and play an important role in regulating blood glucose. Glycogen synthase kinase-3 (GSK-3β) can regulate the activity of glycogen synthase (GS) in the insulin signaling pathway. To evaluate the effects of HNCP on glycometabolism in STZ-induced type 1 diabetic mice, the protein expressions of GK, PEPCK1, G6Pase, and GSK-3β in liver tissues were assessed. As shown in Figure 7, the expression levels of G6Pase and PEPCK1 were significantly up-regulated in the DM group relative to that in the Con group, while the GK and phosphorylation of GSK-3β were significantly down-regulated. After HNCP intervention, the expression levels of G6Pase and PEPCK1 in the liver of the diabetic mice were decreased, while the GK and phosphorylation of GSK-3β were increased. The results indicated that HNCP treatment could significantly improve glucose metabolism disorder in STZ-induced diabetes mice.

## 3. Discussion

Marine by-products such as skin and bone of fish are rich sources of collagen. Marine collagen hydrolysates have demonstrated antioxidant and anti-diabetic activities [28,29]. The amino acid composition and molecular weight of peptides in the hydrolysate were found to be the key factors for antioxidant activity. It was reported that small molecular peptides with 2–20 amino acid residues from marine by-products had the most potent antioxidant activities [30]. Thus, we compared the antioxidant activities of different-molecular-weight components of collagen hydrolysates from *Harpadon nehereus* bones. As expected, the small molecular (Mw < 1 kDa, HNCP) had the highest content and DPPH scavenging activity (42.0% at 5 mg/mL) among all the components with different molecular weights. The peptide fractions < 3 kDa from brown *Lens culinaris* protein hydrolysates showed about 23% of DPPH scavenging rate at a 5 mg/mL concentration, which was significantly lower than that of HNCP [31]. Moreover, the total amino acid compositions showed that HNCP is rich in Gly, Ala, and Pro but low in Phe, Ile, and Tyr. Several studies have shown that peptides containing hydrophobic amino acids such as Phe, Tyrosine, Iso, and Pro exert a higher antioxidant activity [32]. Therefore, the antioxidant activity of HNCP might be predominantly due to the high contents of Pro.

Diabetes is a metabolic disease characterized by high blood glucose levels and metabolic disorders. According to statistics, there were 529 million people living with diabetes worldwide in 2021 [33]. Accumulating evidence shows that the pathological and functional damage of organs induced by diabetes is an important cause of death, while the high level of oxidative stress is closely related to organ damage and dysfunction [34,35]. Thus, antioxidant peptides from marine collagen have gained widespread interest as a potential drug to combat oxidative stress in diabetes patients. In this study, high-dose STZ-induced type 1 diabetes mice were used to study the effect of HNCP on diabetes and the underlying mechanisms. It was observed that a 240 mg/kg HNCP administration decreased the blood glucose levels by 44.5% after 120 min and increased insulin secretion by 142.0% in STZ-induced diabetes mice, which were significantly higher than those by peptides from red deer antlers (about 30%) [36]. In addition to regulating insulin secretion, glycogen synthesis and decomposition are also important ways to regulate blood glucose levels. Our study found that HNCP treatment could increase the synthesis of liver glycogen and muscle glycogen in STZ-induced diabetes mice, indicating that HNCP might improve the glucose tolerance of diabetic mice. This speculation was supported by glucose tolerance tests as shown in Figure 2C. These results indicated that HNCP had a significant therapeutic effect on STZ-induced diabetes mice by improving the insulin level and synthesis of glycogen.

Diabetes often affects liver function, which in turn leads to abnormal lipid metabolism. The study on lipid metabolism showed a higher serum lipid concentration including TG, TC, and LDL-C in STZ-induced diabetes mice than those in the Con group. The levels of TG, TC, and LDL-C were significantly lower in the HNCP-treated mice, whereas the HDL-C levels were higher. In particular, TG decreased by 42.2% after the 240 mg/kg HNCP treatment, while no significant decrease in TG levels was observed in diabetic mice treated with peptides from red deer antlers [36]. These findings suggested that HNCP administration could improve the lipid metabolism disorder of STZ-induced diabetes mice. Similar results were reported in collagen peptides from skate (*Raja kenojei*) skin [37]. We assumed that the blood glucose-lowering effects of HNCP might positively contribute to the lipid levels. The liver is the major organ responsible for glucose and lipid metabolism. Abnormal blood glucose and lipid levels indicate the presence of liver damage. In addition, the insulin level is an important indicator of pancreatic function. Our previous study demonstrated that abnormal blood glucose, insulin, and lipid levels were present in STZ-induced type 1 diabetes mice, and HNCP ameliorated the abnormality of these indicators. Thus, the effects of HNCP on liver and pancreatic damage were evaluated. H&E staining showed that HNCP treatment attenuated the cell swelling and apoptosis in the liver and pancreas. In addition, HNCP supplementation significantly reduced the serum ALT and AST levels in the STZ-induced diabetes mice. ALT and AST are biological indicators of liver pathological changes [38]. High levels of ALT and AST in serum are often accompanied by liver damage [39]. Therefore, these results indicated that HNCP treatment could alleviate hepatic damage in diabetic mice.

In the process of diabetes, hyperglycemia causes a surge of free radicals in the body, which contributes to liver damage. Therefore, oxidative stress is one of the typical pathophysiological features of diabetes. In the present study, lower levels of antioxidant enzymes (SOD, CAT, and GSH-Px) and higher levels of lipid peroxidation product (MDA) were observed in the DM group compared with those in the Con group. However, HNCP administration markedly decreased the MDA level and increased the levels of SOD, CAT, and GSH-Px in STZ-induced diabetes mice. These results indicated that HNCP significantly reduced oxidative stress by increasing the expression of antioxidant enzymes in diabetes mice, thus alleviating liver damage as shown in Figure 4. Our results are consistent with the previous studies wherein the marine peptides mitigated oxidative stress by increasing the levels of antioxidant enzymes such as CAT, SOD, and GSH-Px [40,41,42]. It has been reported that peptides with a high proportion of hydrophobic amino acids tend to have strong ability to enhance antioxidant enzyme activities [43]. Therefore, the reason that HNCP improves the activities of antioxidant enzymes may be related to its high proportion of Pro.

The increase in the expression of antioxidant enzymes can enhance the antioxidant capacity of the body to remove excess free radicals, which are regulated by the upstream signaling molecule Nrf2. Under the intervention of some substances, Nrf2 is dissociated from Keap1 into the nucleus and binds to the antioxidant response elements, resulting in the transcription of antioxidant enzymes. Thus, the level of nuclear Nrf2 (n-Nrf2) is positively correlated with the antioxidant capacity of the body. A large number of studies have reported that Nrf2 activation can effectively suppress intracellular oxidative stress in diabetes and mitigate its complications [44,45,46]. In our study, HNCP treatment significantly increased the n-Nrf2 expression and downstream proteins HO-1 and NQO1 in STZ-induced diabetes mice. Accordingly, we assumed that the attenuating effects of HNCP on oxidative damage in diabetic mice might positively contribute to the activation of Nrf2-mediated antioxidant pathways.

Glucose metabolism is directly related to blood glucose concentration. The above experiments confirm that HNCP can reduce the blood glucose of diabetic mice by increasing the content of liver and muscle glycogen. To further explore the hypoglycemic mechanism of HNCP, the effects of HNCP on the expression of proteins related to glucose metabolisms such as GK, G6Pase, PEPCK1, and GSK-3β were investigated. Numerous studies have shown that GK expression and GSK-3β phosphorylation are generally significantly reduced in the liver of diabetic patients [47], while G6Pase and PEPCK1 are usually significantly increased [48]. Our results showed that the expression levels of G6Pase and PEPCK1 in hepatocytes were significantly increased, but GK and GSK-3β phosphorylation were significantly decreased in STZ-induced diabetic mice. However, HNCP administration significantly improved this phenomenon. This result indicated that HNCP could improve the expression levels of glycogenesis and gluconeogenesis enzymes in diabetic mice, which might be attributed to improvements in blood glucose levels and insulin secretion.

To date, many compounds have been reported to improve diabetes symptoms or complications by mediating oxidative stress and glucose metabolism. Peptides derived from seaweed protein revealed antioxidant and antidiabetic properties [20]. The collagen peptides from *Oreochromis niloticus* skin have also been reported to exhibit antioxidant and hypoglycemic effects [49]. In this study, HNCP, the small peptides (Mw < 1 kDa) derived from the collagen hydrolysate of *Harpadon nehereus* bones, exhibited the antioxidant effect by activating an Nrf2/ARE pathway and a hypoglycemic effect by improving the glucose metabolism in STZ-induced diabetic mice. Further studies are needed to isolate the peptides and identify those sequences, and subsequently, verify the activities of these peptides.

## 4. Materials and Methods

### 4.1. Chemicals and Reagents

*Harpadon nehereus* were purchased from Zhoushan aquatic products market. Papain was purchased from Aladdin Reagent Co., LTD (Shanghai, China). The antioxidant enzymes kits were from Nanjing Jiancheng Bioengineering Institute (Nanjing, China). Streptozocin (STZ) was purchased from Sigma Company (Saint Louis, Missouri, USA). Citrate-sodium citrate buffer and 4% paraformaldehyde were purchased from Ranger Technology Co., LTD (Beijing, China). The insulin ELISA kit was purchased from Wuhan Illarite Biotechnology Co., LTD (Wuhan, China). RIPA lysate was purchased from Biyuntian Biotechnology Research Institute (Shanghai, China). The antibody of β-Actin and horseradish peroxidase (HRP) were purchased from Biyuntian Biotechnology Co., LTD (Shanghai, China). The remaining antibodies were purchased from Proteintech Group, Inc (Wuhan, China). The reagents used in the Western blot were purchased from the reagent supplier Ningbo Hangjing Biotechnology Co., LTD (Ningbo, China).

### 4.2. Preparation of Collagen Peptides from Harpadon nehereus Bone

Under the conditions of pH 8, 55 °C, enzyme dosage of 5500 U/g, and enzymolysis time of 4 h, papain was selected for the enzymolysis of the bone collagen of Harpadon nehereus. The molecular weight (Mw) distribution of polypeptides in the hydrolysate was analyzed by HPLC. Ultrafiltration membranes with interception diameters of 10, 5, 3, and 1 kDa were used sequentially. The filtrate of each part was collected and freeze-dried for the determination of the DPPH free-radical scavenging rate according to the method by Abdelmawgood [50]. Based on the DPPH scavenging rate and content, peptides with Mw < 1 kD (HNCP) were calculated for animal experiments.

### 4.3. Laboratory Animals

Male C57BL/6J mice weighing 18 ± 2 g were fed in the SPF animal laboratory of Zhejiang Ocean University for 7 days under a 12 h dark/light cycle, with free access to food and water. The mice were randomly divided into a control group (Con) and an experimental group according to body weight. Mice in the experimental group were intraperitoneally injected with STZ (55 mg/kg/d) for 5 days to establish the type 1 diabetes model. The fasting blood glucose level of the mice was continuously monitored on the 7th day after the injection. Excluding 2 mice with failed modeling, the remaining mice with successful modeling were randomly divided into 4 groups: diabetic model group (DM, *n* = 8), positive drug group (MET, *n* = 8), low-dose HNCP group (*n* = 7), and high-dose HNCP group (*n* = 7). They were given sufficient drinking water and food, and the bedding material was changed in time. The Con group and the DM group were fed the same amount of distilled water every day. The MET group was given a metformin solution by gavage at a dose of 160 mg/kg, and the low-dose HNCP and high-dose HNCP groups were fed the HNCP solution by gavage at a dose of 80 mg/kg and 240 mg/kg, respectively. Excluding one dead mouse in the DM group, all the mice were euthanized by cervical dislocation after 4 weeks of feeding [51]. Blood was collected from the eyeballs, and tissues such as liver, kidney, epididymis fat, and pancreas were quickly extracted and stored at −80 °C. All animal experiments were carried out in accordance with the guidelines of the Animal Protection and Utilization Committee of the China Animal Protection Commission (No. 2021029).

### 4.4. Measurement of Blood Glucose, Insulin, and Glycogen

Water and fasting were prohibited 8 h before blood glucose measurement. Blood was collected with a disposable needle tail vein for measurement of blood glucose using a blood glucose test paper and blood glucose meter (Bayanjin flagship store). The obtained blood was immediately centrifuged at 4 °C and 8000 r/min for 5 min. Insulin content in the upper serum and glycogen content in the liver/muscle were measured using the mouse insulin enzyme-linked immunosorbent assay kit (NanJing JianCheng Bioengineering Institute, Nanjing, China) and the anthracenone method, respectively [52].

### 4.5. Oral Glucose Tolerance Test (OGTT)

The oral glucose tolerance test (OGTT) was performed according to the method of Wang et al. [53] with minor modifications. After four weeks of administration, the mice fasted overnight during the fifth week. Each mouse was given a 40% glucose solution by gavage at a dose of 2 g/kg. After feeding the glucose, the blood was collected and measured for blood glucose at 0, 30, 60, and 120 min using a blood glucose test paper (Bayanjin flagship store). The blood glucose time curve was drawn, and the area under the curve of blood glucose response (AUC) was calculated.

### 4.6. Determination of Lipid-Related Indexes in Mice

The contents of TG, TC, HDL-C, and LDL-C in the serum were measured using an enzymatic method [54] provided by the kits (TG, TC, HDL-C, and LDL-C kits) (NanJing JianCheng Bioengineering Institute, Nanjing, China).

### 4.7. Histological Evaluation of Liver and Pancreas

Liver and pancreas tissues were fixed with 4% paraformaldehyde for 24–48 h and dehydrated using anhydrous ethanol. The fixed tissues were embedded with paraffin and cut into 3 μM thick sections. The sections were stained with standard hematoxylin-eosin (H&E), and then the morphological changes of the liver and pancreas were captured by a light microscope [55].

### 4.8. Detection of ALT, AST, MDA, CAT, SOD, and GSH-Px Levels in Liver

The liver tissue was ground into homogenate in cold saline and centrifuged at 12,000 r/min for 10 min. The levels of ALT, AST, MDA, CAT, SOD, and GSH-Px in the supernatant were determined by the kits from NanJing JianCheng Bioengineering Institute (ALT, AST, MDA, CAT, SOD, and GSH-Px kits) [56]. The protein concentration in the tissue homogenate was measured using the BAC protein detection kit (NanJing JianCheng Bioengineering Institute, Nanjing, China).

### 4.9. Western Blot

The liver tissue (0.1 g) that had been ground into a fine powder was mixed with the lysate (RIPA lysis buffer: protease inhibitor mixture: phosphatase inhibitor = 50:1:1) and incubated on ice for 30 min with agitation every 6 min. After centrifugation at 12,000 rpm for 10 min, protein was extracted from the cytoplasm and nucleus using a protein extraction kit from NanJing JianCheng Bioengineering Institute (Nanjing, China) [57]. After extraction, the total proteins concentrations in the liver were measured with a BCA protein assay kit (NanJing JianCheng Bioengineering Institute, Nanjing, China). The protein sample was diluted to a suitable concentration with a buffer at a ratio of 1:4. The mixture was boiled in the water bath for 10 min and then centrifuged at 12,000 r/min for 10 min. The supernatant was collected and stored at −80 °C as a standby.

A PVDF membrane was immersed in 5% BSA solution prepared using TBST (containing 0.1% Tween-80) and closed at room temperature for at least 1 h. They were then incubated overnight with the corresponding primary antibody against Nrf2 (1:2000), β-Actin (1:1000), H3 (1:8000), HO-1 (1:2000), NQO1 (1:20,000), GK (1:2000), PEPCK1 (1:10,000), G6Pase (1:2000), GSK-3β (1:20,000), and p-GSK-3β (1:5000) at 4 °C. The membrane was then incubated with horseradish peroxidase (HRP) (1:500)-labeled secondary antibody at room temperature for 1 h. An enhanced chemiluminescence (ECL) kit (NanJing JianCheng Bioengineering Institute, Nanjing, China) was used to detect the strength of specific bands.

### 4.10. Statistical Analysis

All experiments were conducted in parallel 3 times. The software GraphPad Prism8.0 was used for one-way ANOVA. The comparison between groups was performed using the Tukey method, and the results were expressed as Mean ± SD.

## 5. Conclusions

In this study, small-molecule peptides (Mw < 1 kD) were prepared from the bone collagen of *Harpadon nehereus* (HNCP). HNCP showed a remarkable antioxidant activity by activating the Nrf2 pathway to increase the level of antioxidant enzymes such as SOD, CAT, HO-1, GSH-Px, and NQO1. In addition, HNCP significantly increased glucose tolerance and insulin secretion in STZ-induced type 1 diabetic mice, thereby reducing blood glucose levels. HNCP can also improve glucose metabolism in STZ-induced type 1 diabetic mice by regulating the expression levels of glycosynthesis- and gluconeogenesis-related enzymes such as GK, PEPCK1, G6Pase, and GSK-3β. This is the first time of preparing antioxidant and hypoglycemic peptides from marine bone collagen. Our results indicated that HNCP may be a potential diabetes treatment. However, further research into the sequences of peptides with these effects is required.

## Figures and Tables

**Table 1:** Amino acid composition of HNCP.

Amino Acid	Residues/1000 Residues
Aspartic acid (Asp)	47.4
Threonine (Thr)	29.2
Serine (Ser)	34.8
Glutamic acid (Glu)	71.4
Glycine (Gly)	336.2
Alanine (Ala)	117.3
Valine (Val)	26.1
Methionine (Met)	11.6
Isoleucine (Ile)	12.3
Leucine (Leu)	27.8
Tyrosine (Tyr)	4.2
Phenylalanine (Phe)	12.3
Lysine (Lys)	22.0
Histidine (His)	6.0
Arginine (Arg)	37.3
Proline (Pro)	116.0
Hydroxyproline (Hyp)	76.5
Hydroxylysine (Hyl)	11.9

## Data Availability

The data presented in this study are available on request from the corresponding author.
